# Transdifferentiation of human male germline stem cells to hepatocytes *in vivo* via the transplantation under renal capsules

**DOI:** 10.18632/oncotarget.14713

**Published:** 2017-01-18

**Authors:** Zheng Chen, Minghui Niu, Min Sun, Qingqing Yuan, Chencheng Yao, Jingmei Hou, Hong Wang, Liping Wen, Hongyong Fu, Fan Zhou, Zheng Li, Zuping He

**Affiliations:** ^1^ State Key Laboratory of Oncogenes and Related Genes, Renji- Med X Clinical Stem Cell Research Center, Ren Ji Hospital, School of Medicine, Shanghai Jiao Tong University, Shanghai 200127, China; ^2^ Department of Andrology, Urologic Medical Center, Shanghai General Hospital, Shanghai Jiao Tong University, Shanghai 200080, China; ^3^ Shanghai Institute of Andrology, Ren Ji Hospital, School of Medicine, Shanghai Jiao Tong University, Shanghai 200001, China; ^4^ Shanghai Key Laboratory of Assisted Reproduction and Reproductive Genetics, Shanghai 200127, China; ^5^ Shanghai Key Laboratory of Reproductive Medicine, Shanghai 200025, China; ^6^ Department of General Surgery, Suqian people's Hospital, The Affiliated Hospital of Xuzhou Medical University, Jiangsu 223800, China

**Keywords:** human, spermatogonial stem cells, transplantation, in vivo, transdifferentiation

## Abstract

Here we proposed a new concept that human spermatogonial stem cells (SSCs) can transdifferentiate into hepatocytes *in vivo*. We first established liver injury model of mice by carbon tetrachloride to provide proper environment for human SSC transplantation. Liver mesenchymal cells were isolated from mice and identified phenotypically. Human SSC line was recombined with liver mesenchymal cells, and they were transplanted under renal capsules of nude mice with liver injury. The grafts expressed hepatocyte hallmarks, including ALB, AAT, CK18, and CYP1A2, whereas germ cell and SSC markers VASA and GPR125 were undetected in these cells, implicating that human SSCs were converted to hepatocytes. Furthermore, Western blots revealed high levels of PCNA, AFP, and ALB, indicating that human SSCs-derived hepatocytes had strong proliferation potential and features of hepatocytes. In addition, ALB–, CK8–, and CYP1A2– positive cells were detected in liver tissues of recipient mice. Significantly, no obvious lesion or teratomas was observed in several important organs and tissues of recipient mice, reflecting that transplantation of human SSCs was safe and feasible. Collectively, we have for the first time demonstrated that human SSCs can be transdifferentiated to hepatocyte *in vivo*. This study provides a novel approach for curing liver diseases using human SSC transplantation.

## INTRODUCTION

Human hepatocyte transplantation has been regarded as an alternative and effective treatment for acute- and end-stage liver failure and metabolic liver diseases [1–3]. Notably, hepatocyte transplantation has several advantages over orthotopic liver transplantation [4–9]: i) hepatocytes from one donor can be used for more patients; ii) hepatocytes could be transplanted into patients with liver diseases multiple times; iii) the transplanted hepatocytes are able to restore the liver parenchyma of patients directly or promote the native liver regeneration by secreting appropriate factors indirectly; and iv) transplantation of human hepatocytes is technically simple and reversible since the native liver doesn't need to be removed. However, severe shortage of primary human hepatocytes, including the limited number and proliferation of these cells, restricts their applications in treating liver disorders [9, 10]. Human embryonic stem (ES) cells and induced pluripotent stem (iPS) cells have been shown to generate hepatocytes [11–14]. Moreover, transplantation of hepatocytes derived from ES cells and iPS cells could repair liver damage in animal models [15, 16]. Nevertheless, several key issues, e.g., ethic and safety issues associated with human ES cells and virus transduction and tumorigenesis related to human iPS cells [17], have not yet been solved for clinical applications of hepatocytes generated from human pluripotent cells. Meanwhile, adult stem cells derived from bone marrow can be converted into hepatocytes and improve liver injury [18, 19]. However, the identity of these cells converting into hepatocytes remains unclear and controversial [20]. In bone marrow, there are several stem cell populations, including hematopoietic stem cells, mesenchymal stem cells, endothelial progenitor cells, multipotent adult progenitor cells, and side population cells, and it is essential to define which type of stem cells to be the actual cells for liver regeneration [21]. Adversely, subsets of stem cells from bone marrow lead to fibrogenesis within the liver in response to injury [20]. Therefore, it is crucial to seek a readily available cell source from another stem cells and/or extra-hepatic tissues to provide human hepatocytes for cell therapy of liver diseases.

Spermatogonial stem cells (SSCs), a subpopulation of type A spermatogonia in mammalian testis, have great plasticity because of their remarkable pluripotency and transdifferentiation potentials [22]. Numerous studies have reported that SSCs from both mice and human can be dedifferentiated *in vitro* to become ES-like cells which can subsequently differentiate to various cell lineages of all three germ layers [23, 24] , suggesting that SSCs have great applications in regenerative medicine. Furthermore, we and the peers have shown that mouse SSCs could be directly transdifferentiated into prostatic, uterine, skin epithelium, mature hepatocytes, and dopaminergic neurons [25–27]. The transdifferentiation process circumvents the adverse intermediate stage of pluripotency, which is much safer to avoid the formation of teratomas and has advantages in future clinical applications. The transdifferentiation doesn't change host genotype, and human SSCs are not involved in ethical issues. Significantly, we have recently demonstrated that human SSCs can directly transdifferentiate into mature and functional hepatocytes *in vitro* [28], which is one step closer to clinical application. However, it remains unknown whether human SSCs are able to transdifferentiate to hepatocytes *in vivo*.

Stem cells are resided in the niche which is the local microenvironment or niche to regulate their self-renewal and differentiation [29, 30]. It has been suggested that the connective tissues and mesenchymal cells are major regulators of epithelial differentiation [25]. For example, SSCs are unipotent since they can only give rise to sperm within seminiferous tubules of mammalian testis. However, once out of the testicular niche, SSCs can acquire pluripotency to become ES-like cells *in vitro* [22]. Moreover, mesenchymal cells from fetal/neonatal organs can induce mouse SSCs directly to transdifferentiate to prostatic, uterine, and skin epithelium *in vivo* [25]. During liver embryonic development, the adjacent septum transversum mesenchyme and hepatic mesenchyme cells (e.g., stellate cells) secrete a series of growth factors and other factors, including FGF, BMP, HGF, Wnt, TGFβ, and retinoic acid (RA), which are essential for hepatogenesis [31, 32]. Given the importance of the niche for stem cell regulation, we selected hepatic mesenchymal cells to coax SSC transdifferentiation *in vivo*. In this study, we established liver injury model of mice using carbon tetrachloride. Moreover, liver mesenchymal cells and renal capsule provided an appropriate niche for transdifferentiation of human SSCs to hepatocytes *in vivo*. Significantly, neither cellular lesion nor tumor formation was seen in numerous pivotal organs and tissues of recipient mice. This study offers a novel approach to generate human hepatocytes directly from human SSCs *in vivo*, which could have significant applications in treating various kinds of liver diseases.

## RESULTS

### Isolation and identification of liver mesenchymal cells

**Figure 1:**
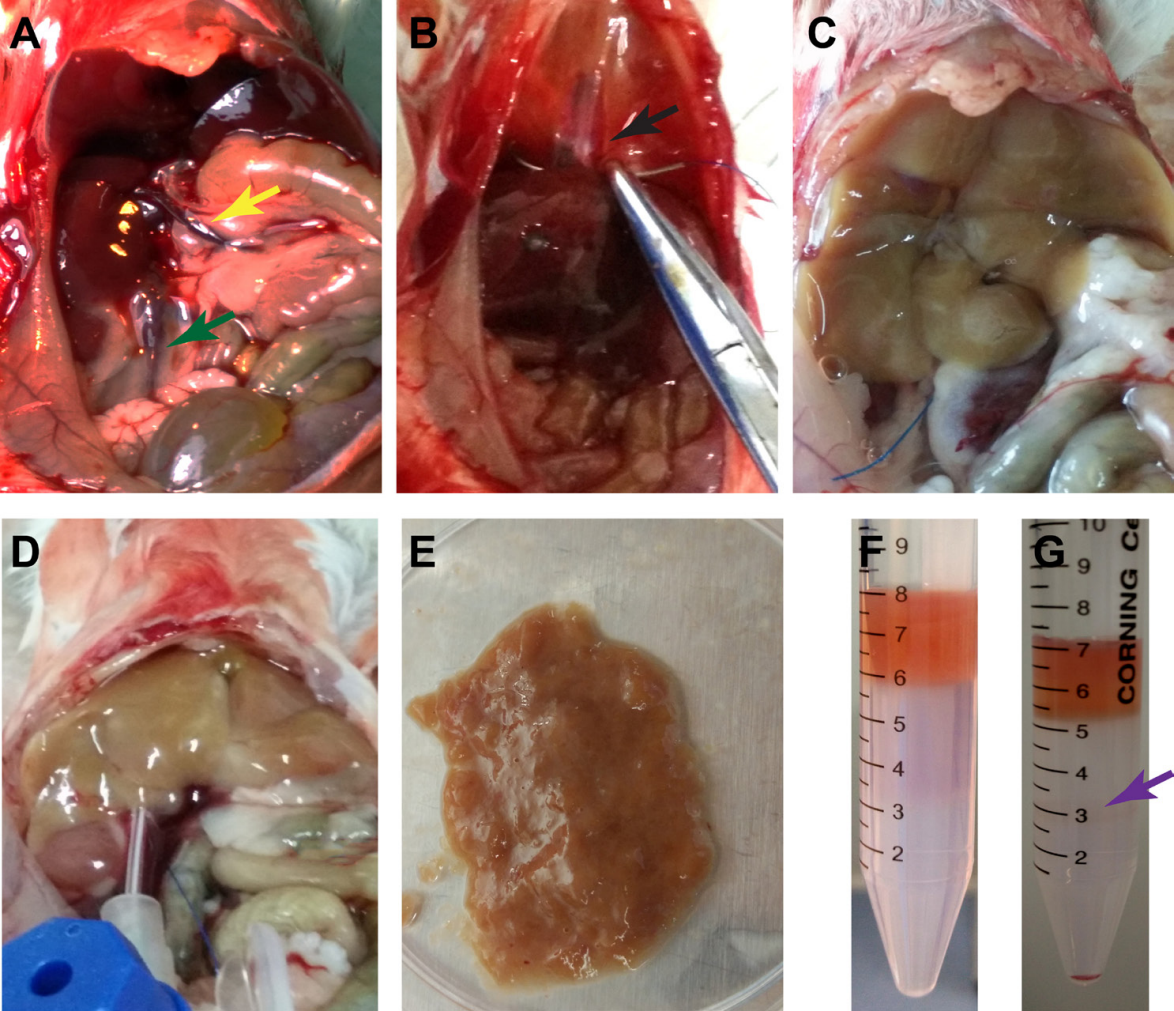
Separation of liver mesenchymal cells from mice (**A**) Exposure of the liver tissues, inferior vena cava (green arrow) and portal vein (yellow arrow) was performed. (**B**) The suprahepatic inferior vena cava (arrow) was sutured. (**C**) Retrograde perfusion was conducted with HBSS buffer via inferior vena cava. (**D**) Sequential perfusion was carried out with pre-warmed pronase E and collagenase IV *in situ*. (**E**) Liver tissues were dissected and minced thoroughly and followed by further digestion with pronase E and collagenase IV *in vitro*. (**F**, **G**) Liver mesenchymal cells (violet arrow) were separated by percoll gradient centrifugation.

**Figure 2:**
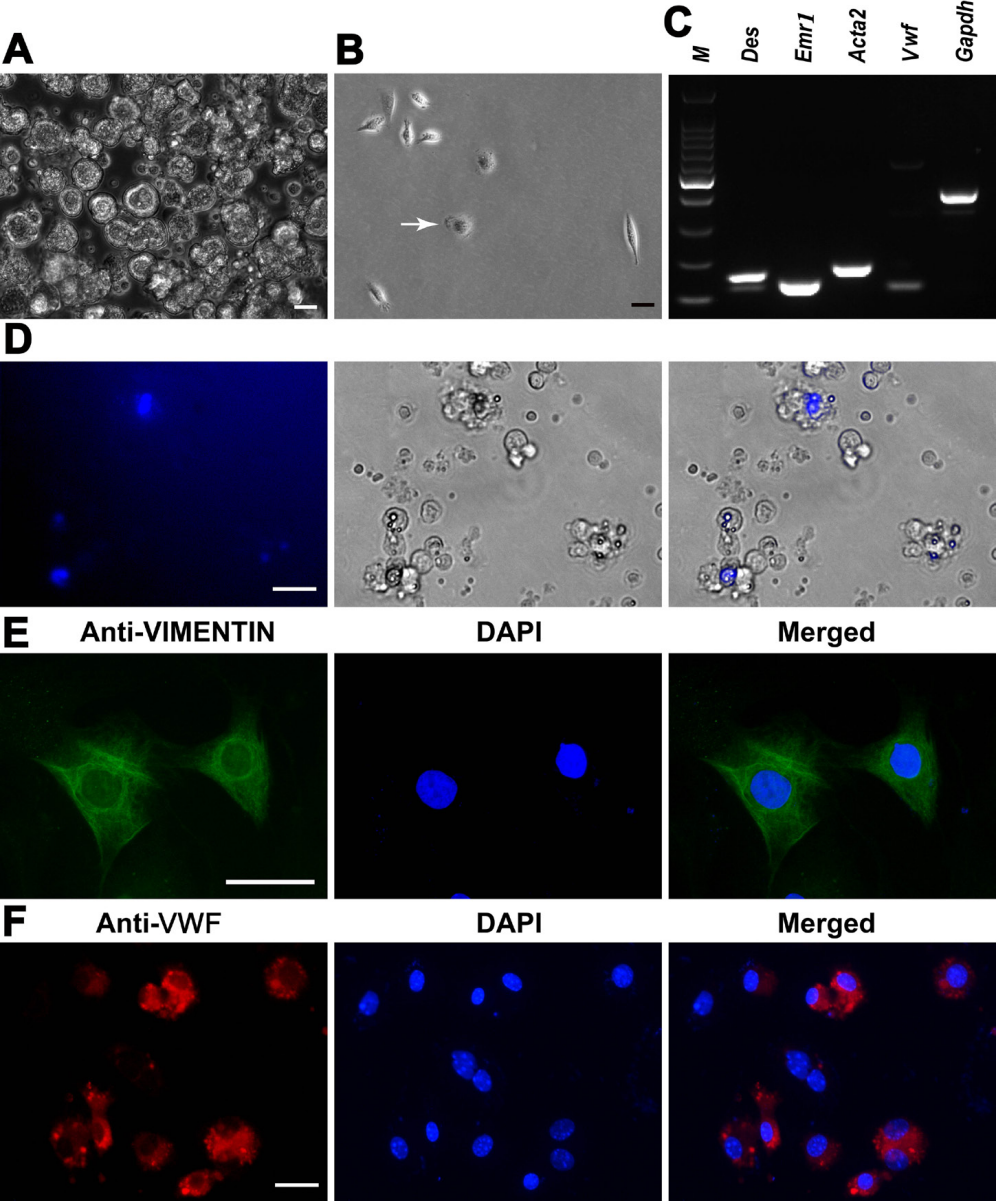
Characterization and identification of mouse liver mesenchymal cells (**A**) Mature hepatocytes were removed by percoll density gradient centrifugation. Scale bar = 10 μm. (**B**) Kupffer cells (white arrow) were isolated by percoll density gradient centrifugation. Scale bar = 10 μm. (**C**) RT-PCR revealed the expression of *Des, Emr1, Acta2*, and *Vwf* in the isolated cells. *Gapdh* served as a loading control of total RNA. (**D**) Lipid droplets and retinoid fluorescence were observed in the freshly isolated hepatic stellate cells. Scale bar = 20 μm. (**E**, **F**) Immunocytochemistry showed the expression of VIMENTIN in hepatic stellate cells (E) and VWF in liver endothelial cells (F) Scale bar in E = 20 μm; scale bar in F = 10 μm.

We first isolated liver mesenchymal cells from mice using retrograde perfusion technique. After anesthetization of mice and a midline abdominal incision, the liver, inferior vena cava, and portal vain were well exposed (Figure 1A). The suprahepatic inferior vena cava was sutured (Figure 1B), which was beneficial for insertion and perfusion. After the perfusion with HBSS buffer, liver tissues were instantly changed from reddish to white (Figure 1C). The liver tissues were sequentially perfused with pre-warmed 0.05% pronase E and 0.05% collagenase IV to delete mature hepatocytes, and they appeared softened after adequate digestion *in situ* (Figure 1D). Liver tissues were carefully removed and minced thoroughly on a Petri dish (Figure 1E), and they were further digested with 0.025% pronase E and 0.025% collagenase IV and followed by 60%-30% percoll gradient centrifugation (Figure 1F) to separate liver mesenchymal cells (interface between 60% percoll and 30% percoll) (Figure 1G) and remove mature hepatocytes (Figure 2A). Liver mesenchymal cells are collected, cultured, and identified by morphology and the expression of genes and proteins. After 6 hours of culture, Kupffer cells were adhered to the culture dishes and they were oval in shape (Figure 2B).

We next analyzed phenotypic characteristics of liver mesenchymal cells at transcriptional and translational levels in order to clarify their identities. As shown in Figure 2C, the freshly isolated cells expressed the transcripts of *Des* (Desmin) and *Emr1* (Emerin homolog 1), markers for hepatic stellate cells, as well as *Vwf* (Von Willebrand factor) and *Acta2* (Actin, alpha 2), hallmarks for endothelial cells and Kupffer cells, respectively. Freshly isolated hepatic stellate cells were identified by highly refractive lipid droplets in the cytoplasm and retinoid fluorescence excited under ultraviolet light (Figure 2D). In addition, immunocytochemistry revealed that more than 90% of the isolated cells were positive for VIMENTIN (Figure 2E) and VWF (Figure 2F), markers for hepatic stellate cells and endothelial cells, respectively, reflecting that the purity of these cells was over 90%. Taken together, these results suggest that the isolated cells were liver mesenchymal cells morphologically and phenotypically.

### Establishment of liver injury model

**Figure 3:**
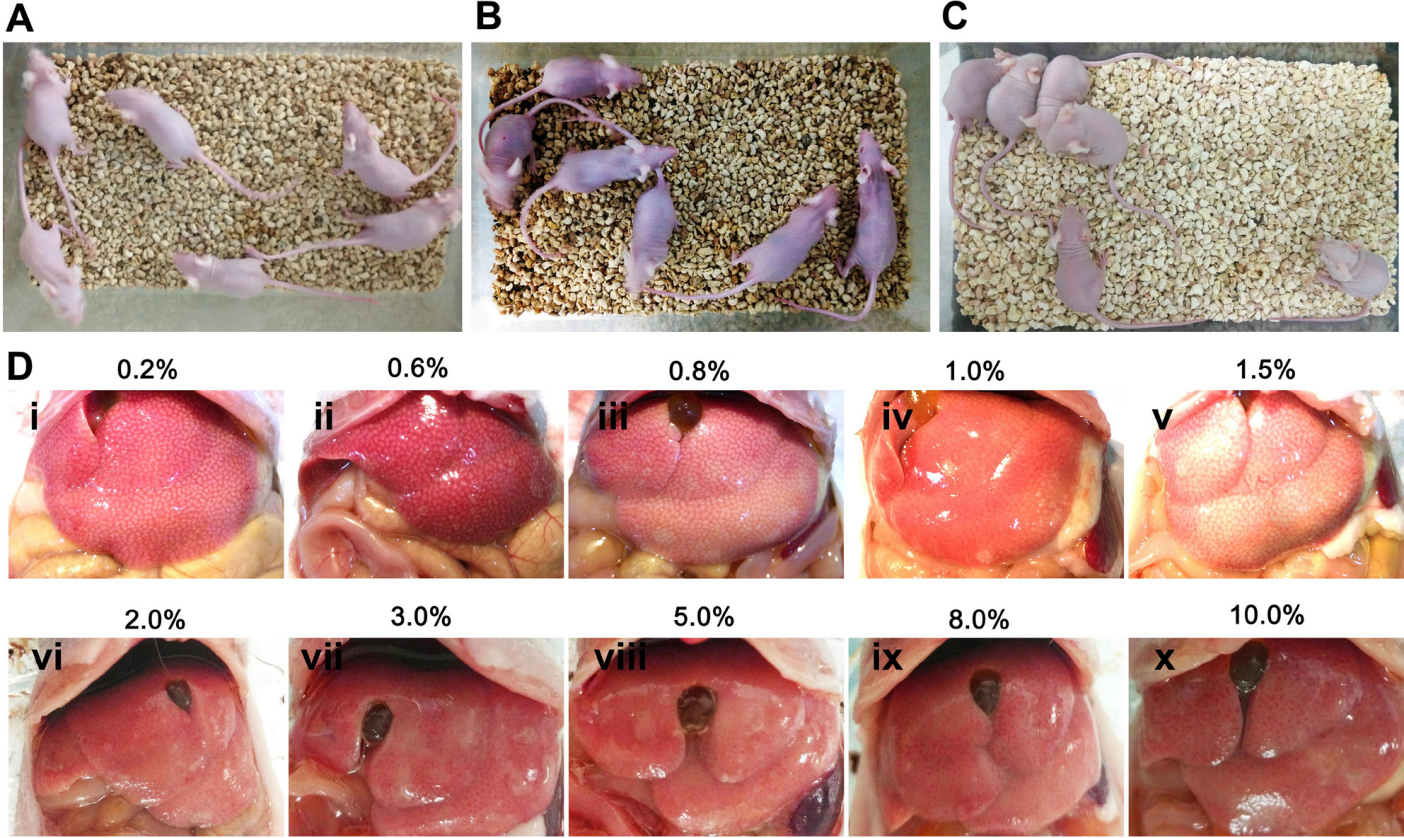
The establishment of mouse liver injury model by carbon tetrachloride (**A**) Nude mice without carbon tetrachloride served as controls. (**B**, **C**) Nude mice were injected with different concentrations (0.2%–10%) of carbon tetrachloride. (**D**) Different levels of liver damage and necrosis by various concentrations (0.2%–10%) of carbon tetrachloride were visible under the macroscope.

To determine the optimal concentrations, a series of concentrations of carbon tetrachloride were utilized, and the levels of liver injury were examined under macroscope and microscope. As shown in Figure 3A–3C, the activities and mental conditions of mice were gradually deteriorated with the concentration increases of carbon tetrachloride . Liver necrosis was visualized and aggravated by the increasing doses of carbon tetrachloride under the macroscope (Figure 3D, i–x).

**Figure 4:**
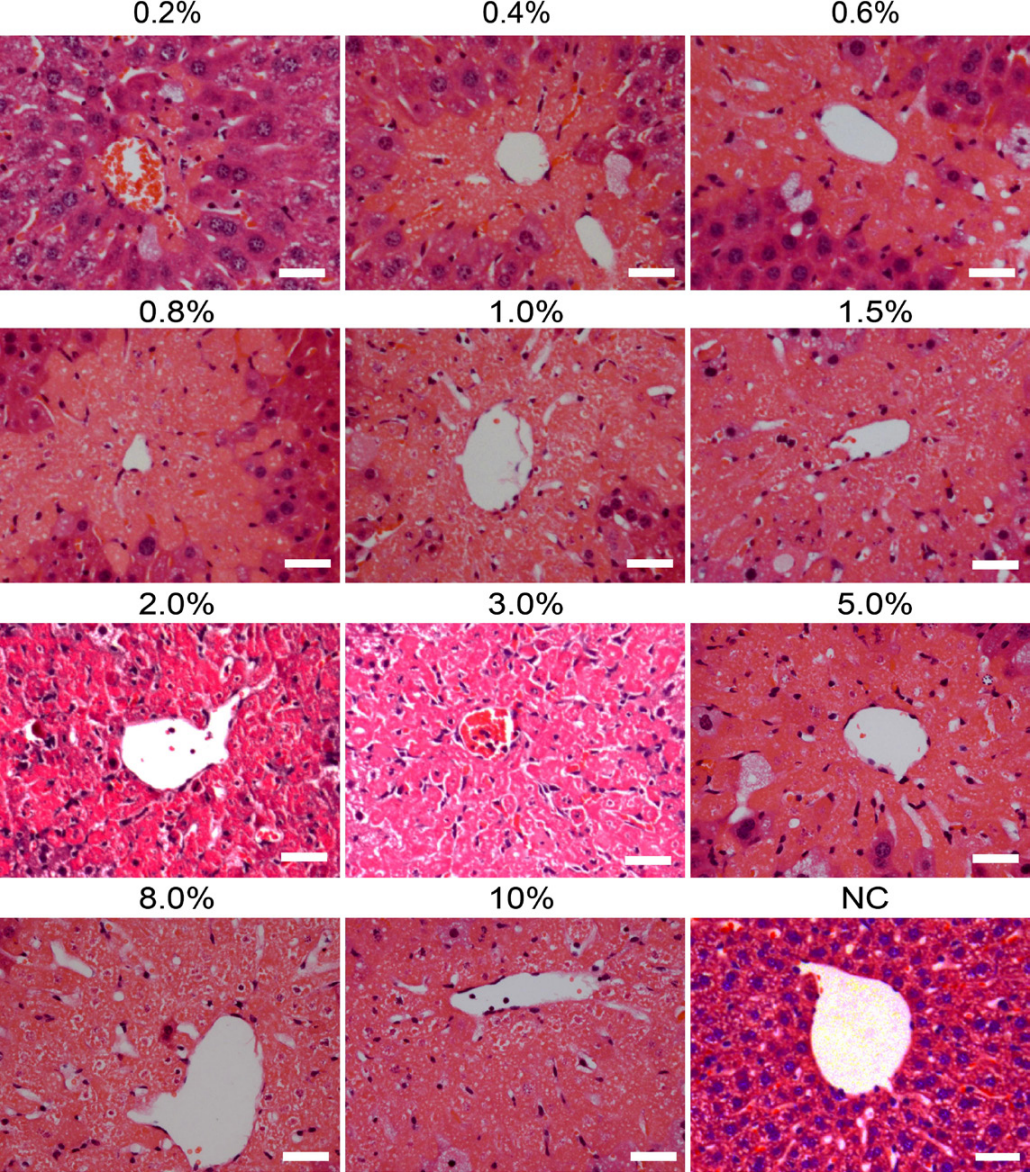
Histological changes in liver tissues of mice induced by different doses of carbon tetrachloride Hematoxylin and eosin (H&E) staining showed the lesion of livers of mice with different concentrations (0.2%–10%) of carbon tetrachloride. Mice treated without carbon tetrachloride were used as a negative control (NC). Scale bars = 50 μm.

To further evaluate the levels of hepatic damage caused by carbon tetrachloride, histological examination was performed using hematoxylin and eosin staining. As shown in Figure 4, carbon tetrachloride led to massive hepatocyte necrosis in liver tissues under microscope. Moreover, the necrosis areas were gradually enhanced with the doses of carbon tetrachloride. Moderate concentrations (1.5%–2.0%) of carbon tetrachloride resulted in 50%-80% of areas with liver lobular damage, while higher doses (e.g., 5%–10%) of carbon tetrachloride caused the death of mice. Therefore, 1.5% of carbon tetrachloride was employed as optimal concentration to establish liver injury model of mice.

### Cell recombination and transplantation of human SSC line under renal capsule of nude mice with liver injury

**Figure 5:**
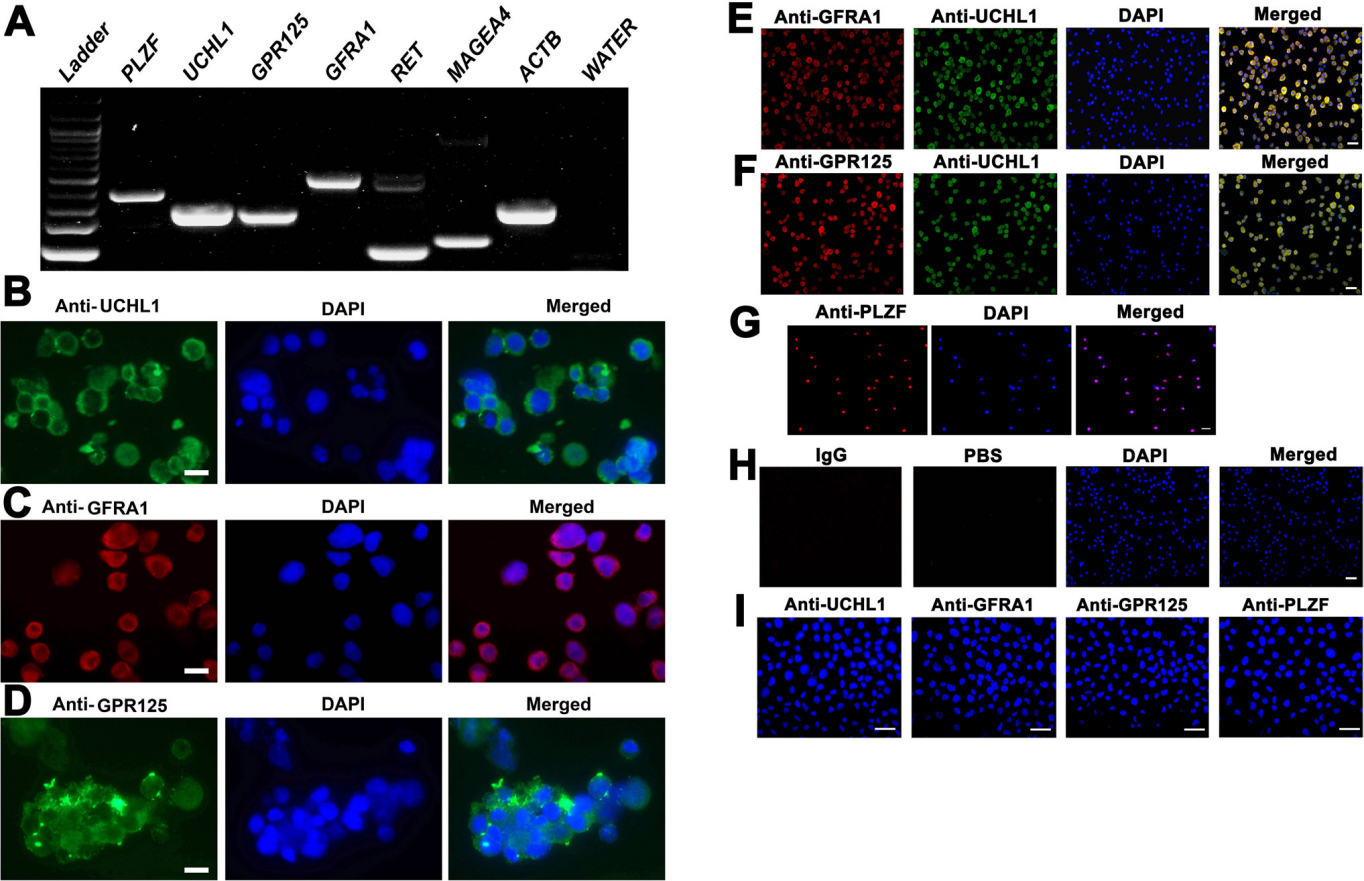
Characterization and identification of human SSC line at transcription and translation levels. (**A**) RT-PCR revealed the expression of *PLZF, UCHL1, GPR125, GFRA1, RET*, and *MAGEA4* in human SSC line. *ACTB* served as a loading control of total RNA, while PCR without cDNA but with water served as a negative control. (**B**–**D**, **G**) Immunocytochemistry showed the expression of phenotypic markers of human SSCs, including UCHL1 (B), GFRA1(C), GPR125 (D), and PLZF (G) in human SSC line. Scale bars in B–D = 10 μm. (E,F,H) Double immunostaining displayed the coexpression of GFRA1 and UCHL1 (**E**), GPR125 and UCHL1 (**F**), as well as isotype IgG and PBS (**H**) in human SSC line. (**I**) Immunocytochemistry illustrated the expression of UCHL1, GFRA1, GPR125 and PLZF in primary human Sertoli cells. Scale bars in E–I = 20 μm.

The cells we utilized in this study were a human SSC line that we established [33]. This human SSC line possesses an unlimited proliferation capacity, and notably it assumes the similar behaviors of primary human SSCs since it is able to colonize, survive and proliferate in the recipient mice after xenotransplantation [33]. We next verified the identity of the human SSC line using various markers for primary human SSCs. RT-PCR revealed that the transcripts of *PLZF*, *UCHL1*, *GPR125*, *GFRA1*, *RET* and *MAGEA4* were detected in human SSC line (Figure 5A). Moreover, immunocytochemistry further showed that UCHL1 (Figure 5B), GFRA1 (Figure 5C), GPR125 (Figure 5D) and PLZF (Figure 5G) were stained positively for this cell line. Double immunostaining demonstrated that human SSC line was co-expressing GFRA1 and UCHL1 (Figure 5E), and GPR125 and UCHL1 (Figure 5F). Replacement of primary antibodies with isotype IgG or phosphate buffer saline (PBS) was used as negative controls, and no specific staining was observed in this cell line (Figure 5H). To rule out the non-specific binding of these antibodies, we performed immunocytochemistry showing that no staining of UCHL1, GFRA1, GPR125 and PLZF was seen in primary human Sertoli cells (Figure 5I). Together, these data implicate that human SSC line we used in the current study was human SSCs phenotypically.

**Figure 6:**
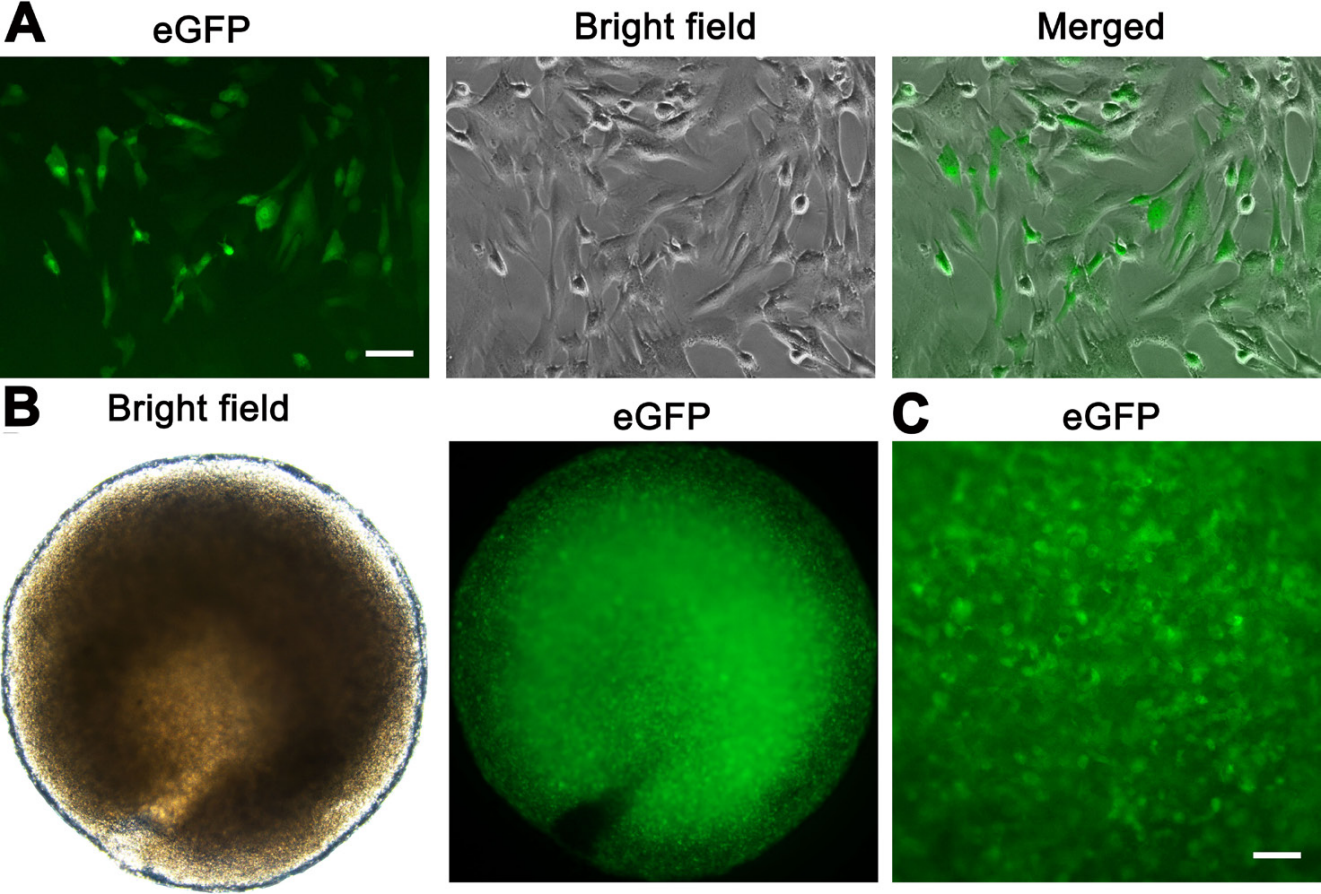
The recombination of human SSC line and mouse liver mesenchymal cells (**A**) The eGFP expression was seen in human SSC line. Scale bar = 20 μm. (**B**, **C**) Cell recombinants including human SSC line and mouse liver mesenchymal cells were incubated in collagen gel. The eGFP fluorescence (B, left panel, and C) was seen in the recombinants showing substantial numbers of human SSC line and a homogeneous distribution. Scale bar in C = 50 μm.

**Figure 7:**
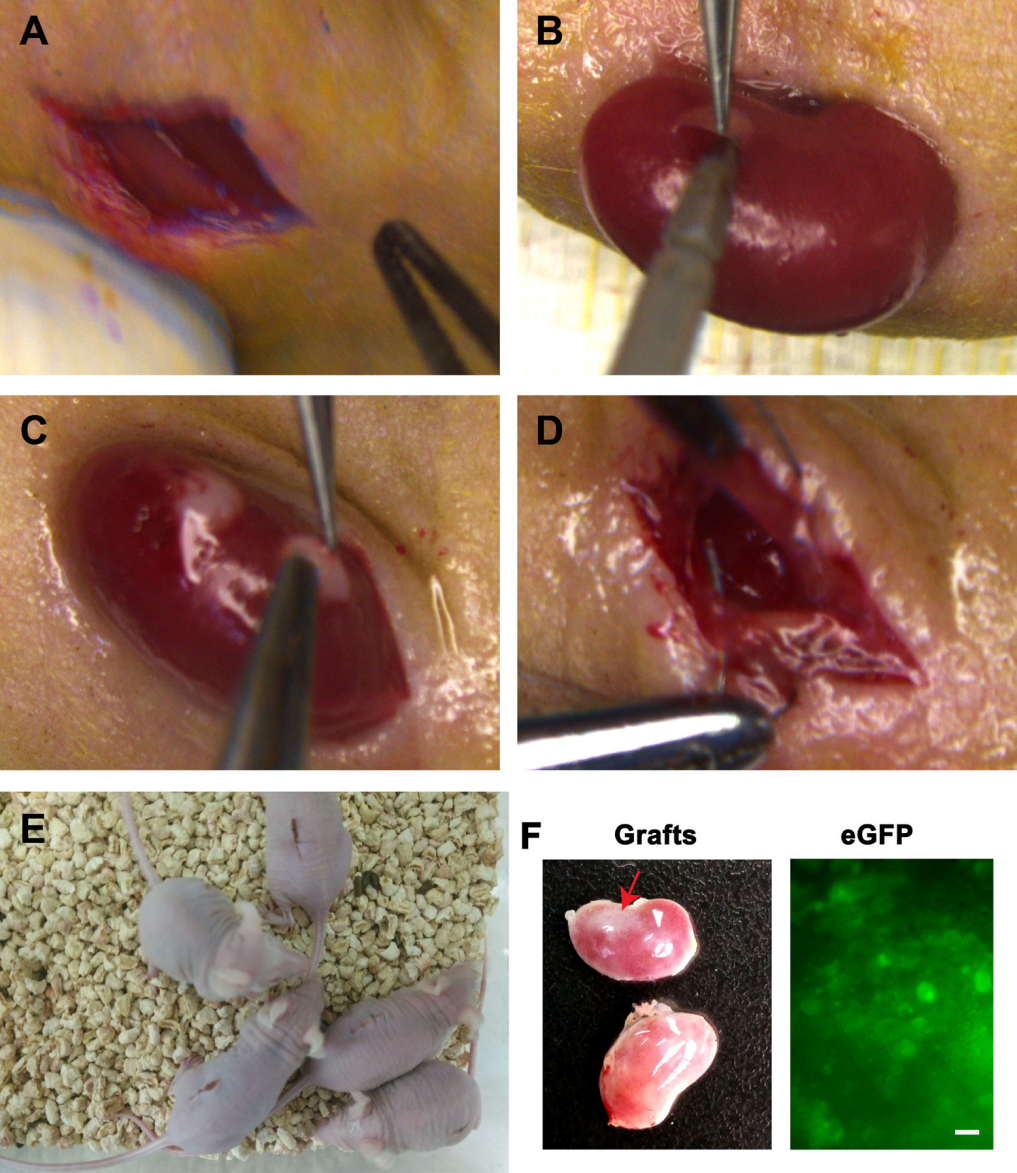
Transplantation of human SSC line and mouse liver mesenchymal cells under the renal capsules of mice (**A**) Skin incision was performed in mouse ventral spine. (**B**) The kidneys of mice were externalized to make tiny incision in the capsules. (**C**) Transplantation of human SSC line and mouse liver mesenchymal cells under renal capsules of mice. (**D**) Kidneys were returned and skin was sutured to prevent backflow of cell recombinants. (**E**) The activities of transplanted mice were checked daily. (**F**) Grafts (red arrow, top kidney) were visible in recipient mice (left panel). The eGFP fluorescence was observed in these grafts (right panel). Scale bar = 20 μm. No graft (low kidney, left panel) was detected in the recipient mice transplanted with human SSC line without liver mesenchymal cells.

The eGFP expression of human SSC line in culture could be observed under a fluorescence microscope (Figure 6A), which was used to track cell origin. Human SSC line was recombined mouse liver mesenchymal cells (Figure 6B), and eGFP expression of human SSC line was clearly seen in cell recombinants (Figure 6B–6C). All cell recombinants were transplanted to the nude mice under the renal capsule (Figure 7A–7D), and the mice with human SSC transplantation were grown well with normal activities (Figure 7E).

### Transdifferentiation of human SSCs into hepatocytes

Four weeks after transplantation, grafts were observed in recipient mice (Figure 7F, left panel). Notably, the grafts derived from human SSC line with liver mesenchymal cells could grow (Figure 7F, top kidney, left panel), whereas there was no graft from human SSCs without liver mesenchymal cells (Figure 7F, low kidney, left panel), which reflects that liver mesenchymal cells plays an important role in regulating the fate determinations of human SSCs. In addition, a number of eGFP-positive cells were detected in these grafts (Figure 7F, right panel), reflecting that the cells were originated from human SSC line.

**Figure 8:**
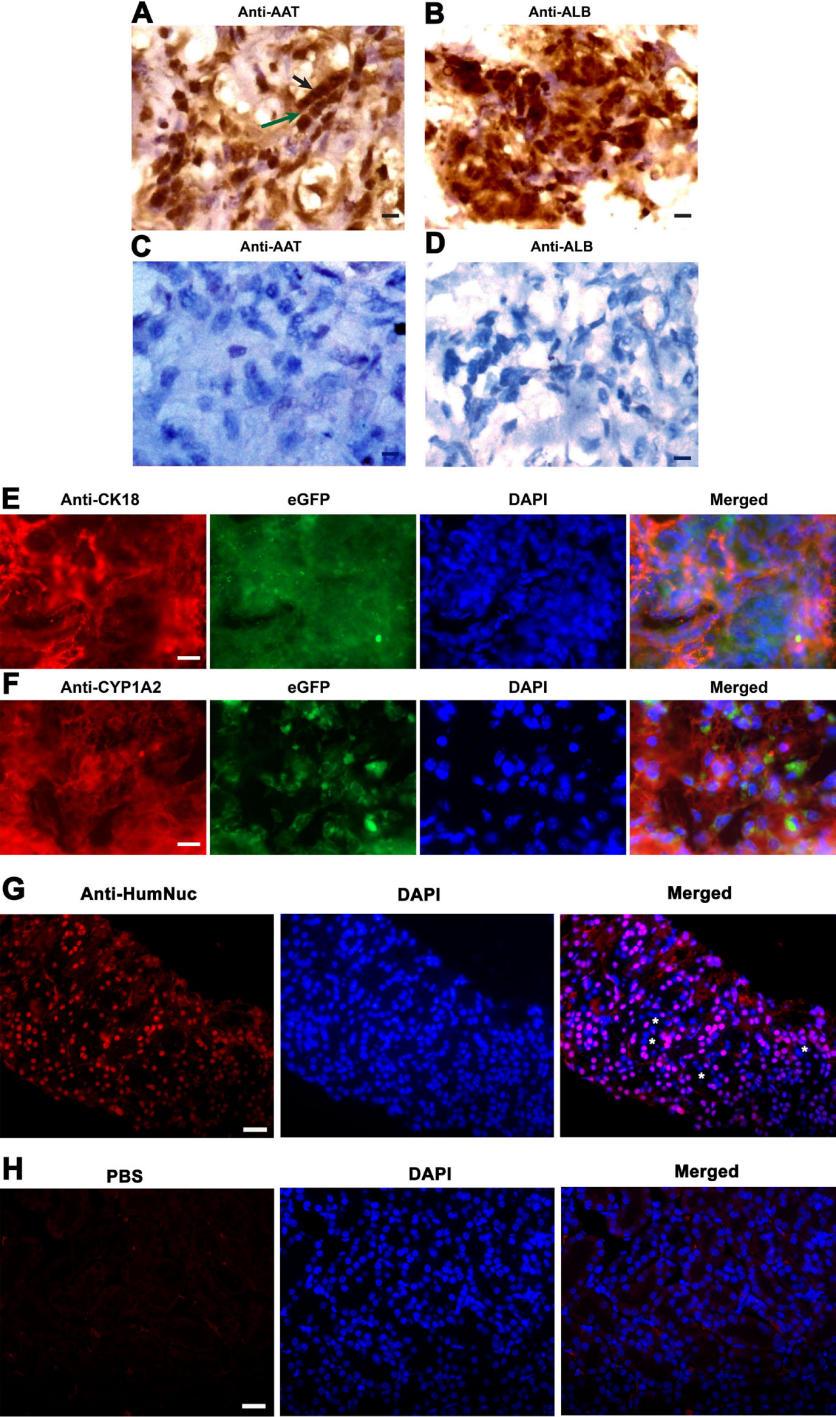
The expression of hepatic markers in the grafts derived from human SSC line and mouse liver mesenchymal cells (**A**–**D**) Immunohistochemistry with DAB staining revealed the expression of AAT and ALB in the grafts derived from human SSC line plus mouse liver mesenchymal cells (A, B) and from human SSC line without liver mesenchymal cells (C, D). Notes: Black arrow in figure A indicated cell cords with tight cell-cell interactions, while green arrow denoted liver plate-like structure. Scale bars in A–D = 10 μm. (**E**, **F**) Immunohistochemistry showed the coexpression CK18 and eGFP (E) as well as CYP1A2 and eGFP (F), and the expression of HumNuc (**G**) in the grafts derived from human SSC line and mouse liver mesenchymal cells. Notes: asterisks denoted HumNuc-negative cells. Replacement with primary antibody with PBS (**H**) was used as a negative control. Scale bars in E and F = 20 μm; and scale bars in G and H = 40 μm.

**Figure 9:**
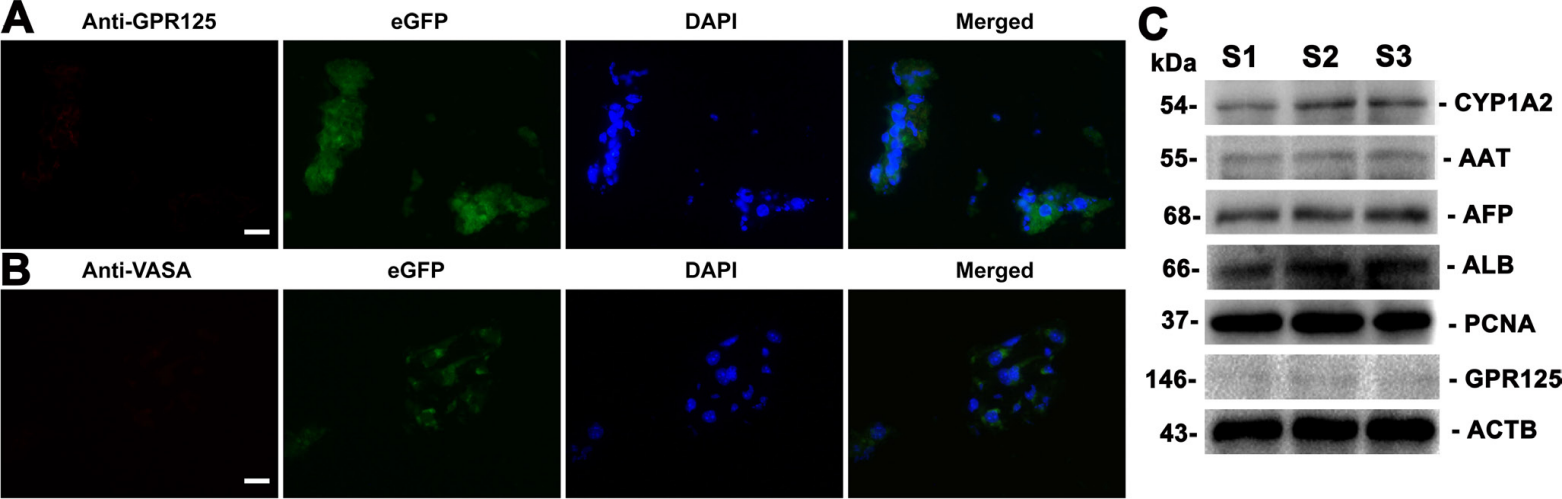
The expression of germ cell and hepatic markers in the grafts derived from human SSC line and mouse liver mesenchymal cells (**A**, **B**) Immunohistochemistry showed the expression of GPR125 (A) and VASA (B) in the grafts derived from human SSC line plus mouse liver mesenchymal cells. Scale bars in A and B = 20 μm. (**C**) Western blots displayed the expression of CYP1A2, AAT, AFP, ALB, PCNA, and GPR125 in the grafts generated from human SSC line and mouse liver mesenchymal cells. Notes: S1, S2 and S3 represented three different samples. ACTB served as a loading control of proteins.

To further determine whether human SSCs transdifferentiate to hepatocytes, we analyzed phenotypic characteristics of the cells derived from the grafts. As shown in Figure 8A–8B, the grafts generated from human SSC line with liver mesenchymal cells were strongly positive for AAT and ALB, specific markers for mature hepatocytes. Notably, a small number of cells formed cell cords with tight cell-cell interactions (Figure 8A), which was similar to liver plate and bile canaliculi structures in mouse liver tissues. In contrast, AAT (Figure 8C) and ALB (Figure 8D) were undetectable in the grafts derived from transplantation of human SSC line without liver mesenchymal cells, suggesting that liver mesenchymal cells established an inductive microenvironment for human SSC commitment to hepatic lineage fate. It remains to be clarified if mouse liver mesenchymal cells could be transdifferentiated to hepatocytes *in vitro* or *in vivo*. To further determine the origin of hepatocytes, we used the combination of hepatocyte markers and eGFP to track cell lineages. As shown in Figure 8E–8F, immunohistochemistry revealed that the grafts expressed hepatocyte specific markers, including CK18 and CYP1A2, and eGFP simultaneously, implicating that these hepatocytes were originated from human SSC line rather than liver mesenchymal cells. Notably, a majority of cells in these grafts were positive for human nuclear antigen (HumNuc) (Figure 8G), and replacement with primary antibody with PBS resulted in no staining (Figure 8H), which further reflects that these cells were indeed originated from human SSC line but not mouse liver mesenchymal cells. Few cells in the grafts were negative for HumNuc (Figure 8G, asterisks), reflecting these cells might be derived from mouse liver mesenchymal cells. Moreover, GPR125 and VASA, hallmarks for human SSCs and germ cells, respectively, were undetected in the grafts from human SSC line with liver mesenchymal cells (Figure 9A–9B), implicating that human SSCs completely converted to hepatocytes.

**Figure 10:**
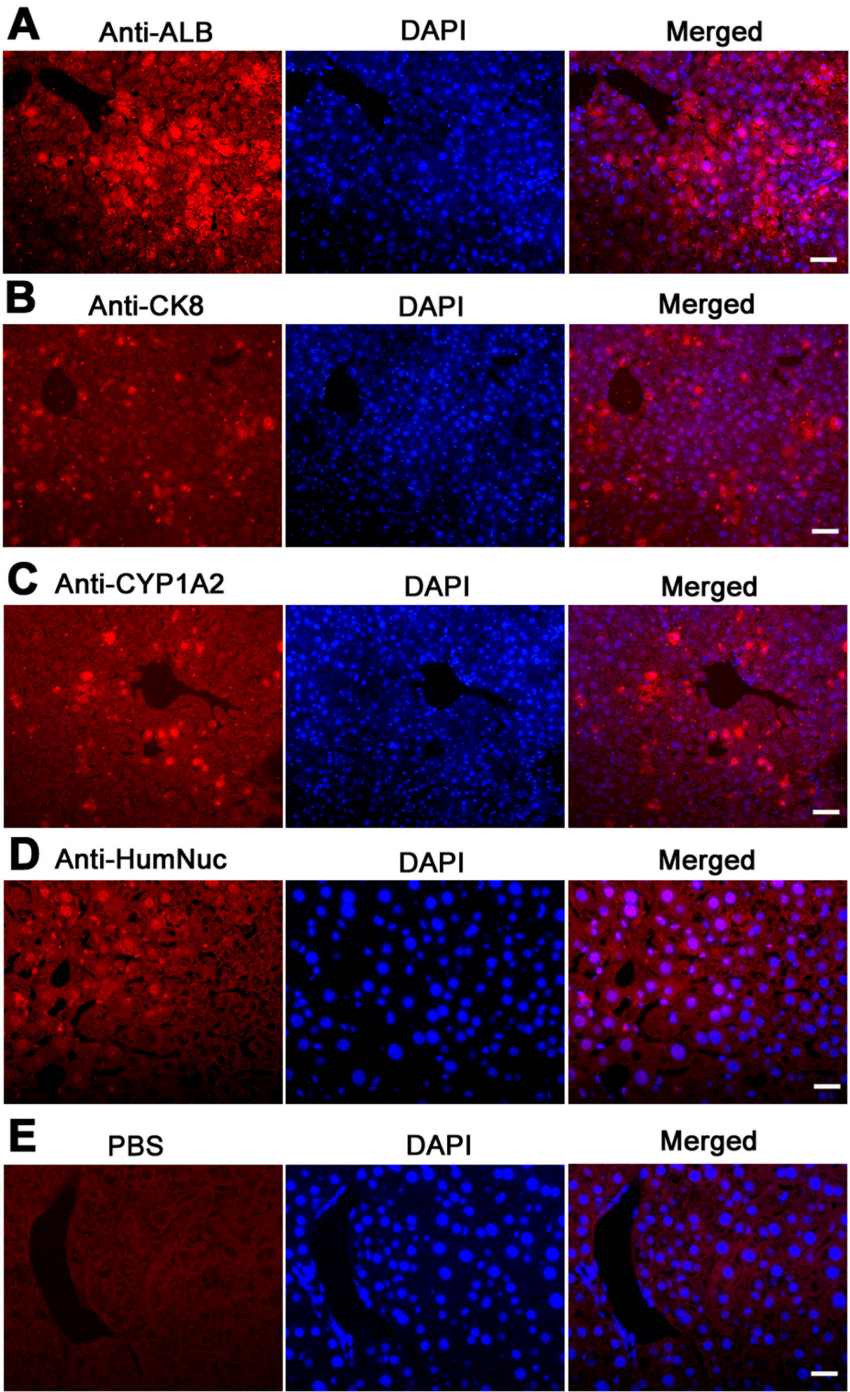
The expression of ALB, CK8, and CYP1A2 in graft hepatic tissues of recipient mice Immunohistochemistry showed that the expression of ALB (**A**), CK8 (**B**), CYP1A2 (**C**), HumNuc (**D**), and PBS (**E**) in graft liver tissues of recipient mice. Scale bars in A–E = 20 μm.

Western blots further demonstrated that CYP1A2, AAT, AFP, ALB, and PCNA were expressed in the grafts derived from human SSC line with liver mesenchymal cells, whereas GPR125 was undetectable in these grafts (Figure 9C), which was completely consistent with our data using immunohistochemistry. Moreover, immunohistochemistry revealed that a number of cells were positive for ALB (Figure 10A), CK8 (Figure 10B), and CYP1A2 (Figure 10C) in liver tissues of recipient mice. Significantly, numerous cells in liver tissues of recipient mice were stained positively for HumNuc (Figure 10D), and replacement with primary antibody with PBS resulted in no staining (Figure 10E), suggesting that these cells were derived from human SSC line. Considered together, our results implicate that human SSCs could be transdifferentiated to hepatocytes phenotypically *in vivo*.

### Safety evaluation of human SSC transplantation

**Figure 11:**
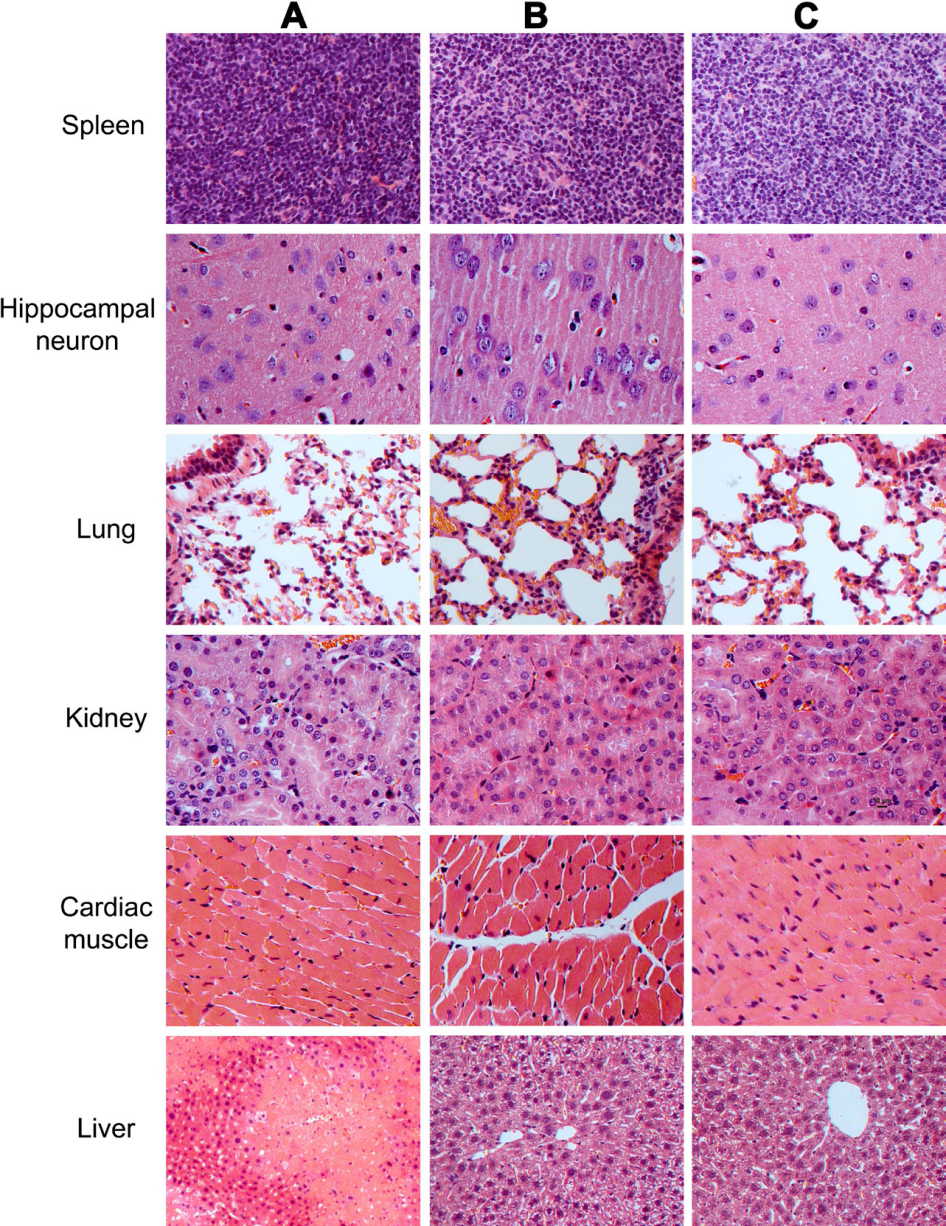
The histological observations of the organs and tissues in mice transplanted with human SSC line and mouse liver mesenchymal cells, mice with liver injury, and the normal nude mice Notes: organs and tissues from mice with liver injury by carbon tetrachloride induction (**A**), as well as organs and tissues from mice at 4 weeks after transplantation of human SSC line and mouse liver mesenchymal cells (**B**), and organs and tissues from normal nude mice (**C**).

To evaluate the safety of human SSC transplantation, we examined the lesion of several pivotal organs and tissues, including brain, lung, kidney, spleen, cardiac muscle, and liver of recipient mice. As shown in Figure 11B, no obvious lesion or teratomas was observed in the organs and tissues of recipient mice transplanted with human SSC line, which was similar to findings in control nude mice (Figure 11C). The results suggest that the transplantation of human SSCs was safe and feasible. Moreover, significant improvement was seen in the liver tissues of mice with human SSC line compared to liver injury mice without transplantation of human SSC line (Figure 11A), which implicates that human SSC line transplantation could ameliorate the liver injury.

## DISCUSSION

We have demonstrated that human SSC line could be directly transdifferentiated to hepatocytes after transplantation with liver mesenchymal cells under renal capsule of mice with liver injury. Notably, this was the first report showing that human SSCs can be transdifferentiated to hepatocytes *in vivo* when placed in a proper microenvironment. The grafts derived from human SSCs expressed specific markers of hepatocytes, including ALB, AAT, CK18, and CYP1A2, reflecting potential functional activity of these cells. In addition, high level of PCNA expression was found in the cells derived from human SSCs, which indicates that these cells had a strong proliferation capacity and offers an invaluable source of human hepatocytes for curing liver diseases. Recently, we have revealed that human primary SSCs can efficiently be transdifferentiated to mature hepatocytes *in vitro* by the conditioned-medium [28]. Significantly, the protocol using human SSC transplantation in this study was more simplified without the intermediate stages *in vitro*, which has advantages to cure patients more timely because liver failure is a severe and rapidly progressive disease. Meanwhile, no obvious lesion or teratomas was observed in a number of important organs and tissues, reflecting that cell-based therapy using the transplantation of human SSCs for liver disorders could be harmless to patients and practicable.

Hepatocytes transplantation has emerged as a promising treatment for a variety of liver diseases [1–3]. However, there is severe shortage of primary human hepatocytes, which precludes their wider clinical applications [9, 10]. Therefore, it is imperative to generate functional hepatocytes from stem cells and/or extra-hepatic tissues for treating patients with liver disorders. A number of studies have shown that hepatocytes can be derived from the ES cells and the iPS cells [11–14]. Nevertheless, a series of obstacles, e.g., ethical issues, tumorigenesis and safety, restrict their clinical use [17]. Here we revealed a novel cell source of human hepatocytes derived from human SSCs, which offers a new therapeutic methodology with distinct advantages over ES and iPS cells. In addition, the transdifferentiation of human SSCs to hepatocytes *in vivo* was relatively simple, which circumvents the lengthy process associated with the differentiation of ES cells and iPS cells *in vitro*. Liver mesenchymal cells might provide a crucial induction microenvironment or niche for the transdifferentiation of human SSCs to hepatocytes *in vivo*, as evidenced by our immunohistochemistry and Western blotting results showing that hepatocytes markers (e.g., ALB, AAT, CK18, and CYP1A2) and HumNuc were strongly expressed in the grafts derived from transplantation of human SSC line and liver mesenchymal cells whereas germ cell and SSC hallmarks (e.g., VASA and GPR125) were undetectable in these grafts. Additionally, the expression of AAT, ALB and CYP1A2 was undetectable in the grafts without recombination of liver mesenchymal cells, further implicating that liver mesenchymal cells (i.e., hepatic stellate cells, endothelial cells, and Kupffer cells) were required for the transdifferentiation of human SSCs to hepatocytes *in vivo*. Notably, we found that numerous cells were stained positively for ALB, CK8, CYP1A2, and HumNuc in hepatic tissues of recipient mice, reflecting that human SSCs could be converted to hepatocytes that might be transferred to liver tissues via blood circulation to repair the damaged liver.

Liver mesenchyme cells can produce a number of factors, including FGF, BMP, HGF, Wnt, TGFβ and RA, which are critical for liver development [31]. These factors can form a complex network helpful for fate decisions of human SSCs, which can't be completely reproduced *in vitro*. Liver mesenchymal cells are able to mimick the environment for liver development, and they might provide an appropriate niche, which generated necessary signals and cell-cell interactions for the transdifferentiation of human SSCs to hepatocytes. This could be verified by previous findings showing that mesenchymal cells are essential for inducing the differentiation of mouse SSCs to cell lineages of skin and prostate [25].

We set up liver damage model of mice by carbon tetrachloride to offer the conversion environment for human SSCs. Carbon tetrachloride can cause injury of specific liver lobular zones and it has been used to establish liver damage model [34]. Although hepatic oval cells have been shown to respond to carbon tetrachloride [34], the internal environment using carbon tetrachloride as a reprogramming approach has not been reported. We have revealed that 1.5% of carbon tetrachloride effectively led to massive injury of liver tissues and induced the transdifferentiation of human SSCs to hepatocytes. A cascade of factors might be released and activated by carbon tetrachloride, which provides an internal environment for liver repair and regeneration by human SSCs. It remains to be determined whether human SSCs could be transdifferentiated into hepatocytes in normal recipient mice.

Human SSCs have significant applications in both reproductive and regenerative medicine because of their unique and great potentials [24, 28]. However, basic studies and clinical usage were seriously hampered due to a limited number of human SSCs and difficulty to obtain human testicular tissues [33]. To solve the urgent problems, we have for the first time established a stable human SSC line with an unlimited proliferation potential by overexpressing SV40 large T antigen [33]. This human SSC line has the similar behaviors of primary human SSCs because it can colonize and proliferate in the recipient mice after xenotransplantation [33]. In the current study, we verified the identity of human SSC line as human SSCs at both transcription and translation levels since it expressed a number of markers for human SSCs. Another advantage of this human SSC line was its eGFP reporter gene as confirmed in this study, which was convenient for tracking cell lineage origin. Therefore, human SSC line was selected to obtain sufficient number of human SSCs for transplantation. Hepatocyte specific markers and eGFP were simultaneously detected and completely co-expressed in the grafts. Furthermore, a number of cells in liver tissues of recipient mice were positive for HumNuc, thus excluding the possibility of regeneration of liver derived from mouse liver mesenchymal cells. These results clearly demonstrated that hepatocytes were originated from human SSCs but not conversion from liver mesenchymal cells. Together, these data illustrate that human SSC line has great potentials and applications, since they have the abilities of transdifferentiation although these cell lines might be unable to differentiate to spermatids and its safety needs to be evaluated because of the overexpression of SV40 large T antigen. It seems to be interesting to determine whether human SSCs could be transdifferentiated to liver mesenchymal cells.

In summary, we have for the first time demonstrated that human SSCs were able to transdifferentiate to hepatocytes *in vivo*. Liver mesenchymal cells were effective for inducing the generation of hepatocytes from human SSCs, and transplantation of human SSCs can significantly improve liver injury with high safety. Therefore, this study offers a novel approach for treating liver diseases based on human SSC transplantation.

## MATERIALS AND METHODS

### Human SSC line and cell culture

Human SSC line was established by transfecting human SSCs with a plasmid (Lenti-EF1α -SV40LargeT-IRES-eGFP) expressing the SV40 large T antigen under the control of the EF1α promoter, and eGFP was utilized as a reporter gene [33]. The identity of human SSC line was evaluated by the expression of genes and proteins for human SSCs using RT-PCR and immunocytochemistry, respectively. Human SSC line was cultured with Dulbecco's Modified Eagle's Medium/Nutrient Mixture F12 (DMEM/F12, Gibco Laboratories, Grand Island, NY) supplemented with 10% fetal bovine serum (FBS, Gibco) and 100 unit/ml penicillin and streptomycin (Invitrogen). The cells were passed every 3-4 days using 0.05% trypsin and 0.53 mM EDTA (Invitrogen) and maintained at 34°C in a humidified 5% CO_2_ incubator.

### Ethics statement

All methods were carried out in accordance with relevant guidelines and regulations of the Institutional Ethical Review Committee of Ren Ji Hospital. All experimental protocols were approved by the Institutional Ethical Review Committee of Ren Ji Hospital, School of Medicine, Shanghai Jiao Tong University (license number of ethics statement: 2012–01). An informed consent of testis tissues used for research only was obtained from each obstructive azoospermia patient.

This study on nude mice was approved by The Institutional Ethical Review Committee of Ren Ji Hospital, and the experiments were carried out strictly in accordance with the care and use of laboratory animals and the related ethical regulation of Ren Ji Hospital, School of Medicine, Shanghai Jiao Tong University.

### Isolation and characterization of mouse liver mesenchymal cells

Liver mesenchymal cells were isolated from BALB/c mice (Shanghai Laboratory Animal Center, Chinese Academy of Sciences, Shanghai, China) of 12 weeks old using retrograde perfusion via the inferior vena cava (IVC) [35]. Briefly, mice were anesthetized and perfused with pre-warmed solutions, including: i) 100 ml HBSS buffer containing 19 mg EGTA (Sigma-Aldrich), 357 mg HEPES (Sigma-Aldrich), and 0.5% penicillin and streptomycin for 20 min, ii) 0.05% pronase E (Sigma) solution containing 100 ml HBSS, 357 mg HEPES and 50 mg pronase E for 7 min, and iii) 0.05% collagenase IV (Gibco) solution containing 100 ml HBSS, 357 mg HEPES, and 50 mg collagenase IV for 5 min. After the *in situ* digestion, liver tissues were carefully excised and minced thoroughly, and they were further digested with pre-warmed 0.025% pronase E and 0.025% collagenase IV *in vitro* for 15 min. Cell suspension was collected and purified by 60% and 30% percoll (Sigma-Aldrich) density gradient centrifugation [35]. The freshly isolated liver mesenchymal cells were identified by morphology and biochemical phenotypes.

### RNA extraction and reverse transcription-polymerase chain reaction (RT-PCR)

Total RNA was extracted from human SSC line and the freshly isolated liver mesenchymal cells using Trizol (Invitrogen, Carlsbad, CA, USA). DNase I was used to remove potential contamination of genomic DNA. Reverse transcription (RT) was performed using First Strand cDNA Synthesis Kit (Thermo Scientific) and PCR was performed according to the protocol described previously [36]. The primer pairs of selected genes, including *PLZF*, *UCHL1*, *GPR125*, *GFRA1*, *RET*, *MAGEA4*, *ACTB*, *Des*, *Emr*, *Acta2*, *Vwf*, and *Gapdh* were designed and listed in Table 1. The PCR reaction started at 94°C for 5 min and was performed as follows: denaturation at 94°C for 30 sec, annealing at specific temperature (Tm) as indicated in Table 1 for 45 sec, and elongation at 72°C for 45 sec. After 35 cycles, the samples were extended at 72°C for additional 10 min. The PCR products were separated by electrophoresis on 1.5% agarose gels, and images were captured by chemiluminescence (Chemi-Doc XRS, Bio-Rad, Hercules, CA). PCR samples without cDNA but with water served as a negative control.

**Table 1:** The primer sequences of genes used for RT-PCR

Genes	Primer sequence		Product size (bp)	Tm (°C)
*Des*	Forward	CAACCTTCCTATCCAGACCTTC	141	60
	Reverse	GTAGCCTCGCTGACAACCTC		
*Acta2*	Forward	AATGGCTCTGGGCTCTGTAA	152	58
	Reverse	CTCTTGCTCTGGGCTTCATC		
*Vwf*	Forward	TGCCTCAGTGGGAGAAAGAT	108	58
	Reverse	CAGGTTTGTGCTCTGCTTGA		
*Emr1*	Forward	CTGCACCTGTAAACGAGGCTT	127	60
	Reverse	GCAGACTGAGTTAGGACCACAA		
*ACTB*	Forward	CGCACCACTGGCATTGTCAT	200	55
	Reverse	TTCTCCTTGATGTCACGCAC		
*UCHL1*	Forward	CCAATGTCGGGTAGATGA	244	55
	Reverse	CCAATGTCGGGTAGATGA		
*GPR125*	Forward	TACCCTTTGGACTTGGTT	246	49
	Reverse	TACCCTTTGGACTTGGTT		
*GFRA1*	Forward	CCAAAGGGAACAACTGCCTG	410	58
	Reverse	CGGTTGCAGACATCGTTGGA		
*PLZF*	Forward	CGGTTCCTGGATAGTTTGC	317	54
	Reverse	GGGTGGTCGCCTGTATGT		
*MAGEA4*	Forward	CCGAGTCCCTGAAGATG	155	50
	Reverse	CAGGACGATTATCAGAAGG		
*RET*	Forward	CCAATGTCGGGTAGATGA	126	52
	Reverse	CCAATGTCGGGTAGATGA		
*Gapdh*	Forward	GCCCTCCCTACTCTCTTGAATA	383	58
	Reverse	AGGAGGGCTGGGTACAATTA		

### Immunocytochemistry

Human Sertoli cells were isolated from obstructive azoospermia patients with normal spermatogenesis using a two-step enzymatic digestion and followed by differential plating. Immunocytochemistry was performed to determine the identity of human SSC line, primary human Sertoli cells, and mouse liver mesenchymal cells pursuant to the procedure described previously [37]. Briefly, cells were fixed in 4% paraformaldehyde (PFA) for 30 min and followed by permeabilization with 0.4% Triton X-100 for 15 min and blocking in 1% bovine serum albumin (BSA, Sigma-Aldrich) for 1 h. The primary antibodies were as follows: UCHL1 (AbD Serotec), GFRA1 (Santa Cruz), GPR125 (Abcam), PLZF (Santa Cruz), VIMENTIN (Abcam), and VWF (Santa Cruz). After incubation overnight at 4°C, rhodamine-conjugated or FITC-conjugated IgG was used as secondary antibodies. The dilutions, resources, and companies of primary and secondary antibodies for immunocytochemistry were shown in Table 2. Double immunostaining was also performed to verify the identity of human SSC line antibodies against GFRA1 and UCHL1 as well as GPR125 and UCHL1. Replacement of primary antibodies with isotype IgG or PBS served as a negative control. The nuclei of cells were stained with DAPI (4′-6-diamidino-2-phenylindole) and the epifluorescence were examined under fluorescence microscope (Nikon Eclipse Ti-S, Nikon Corporation, Tokyo, Japan).

**Table 2:** The antibodies used for immunostaining and Western blots

Antibodies	Dilutions	Resources	Companies
UCHL1	ICC: 1:200	Rabbit	AbD Serotec
GFRA1	ICC: 1:200	Goat	Santa Cruz
GPR125	ICC: 1:200	Rabbit	Abcam
	WB:1:100		
	IHC:1:100		
VASA	IHC:1:100	Goat	Santa Cruz
CK18	IHC:1:100	Mouse	Santa Cruz
CK8	IHC:1:100	Goat	Santa Cruz
AFP	WB:1:100	Mouse	Santa Cruz
ALB	IHC:1:100	Mouse	Santa Cruz
	WB:1:100		
AAT	IHC:1:100	Goat	Bethyl
	WB:1:100		
CYP1A2	IHC:1:100	Rabbit	Santa Cruz
	WB:1:100		
PCNA	WB:1:500	Rabbit	Abcam
VWF	ICC: 1:200	Mouse	Santa Cruz
DESMIN	ICC: 1:200	Mouse	Abcam
VIMENTIN	ICC: 1:200	Mouse	Abcam
ACTB	WB: 1:5000	Mouse	Proteintech
AlexFlour488	1:500	Goat-anti-Rabbit	Invitrogen
AlexFlour594	1:500	Donkey-anti-Goat	Invitrogen
AlexFlour647	1:500	Goat-anti-Mouse	Invitrogen
AlexFlour594	1:500	Goat-anti-Mouse	Invitrogen
HRP-conjugated IgG	1:1000	Goat anti-Mouse	Santa Cruz
HRP-conjugated IgG	1:1000	Goat anti-Rabbit	Santa Cruz

### Establishment of mouse liver injury model

Carbon tetrachloride was utilized to established liver injury model of nude mice [34]. In order to provide a proper internal environment, different concentrations of carbon tetrachloride (Sinopharm Chemical Reagent Co., Ltd, Shanghai, China), ranging from 0.2% to 10% diluted in olive oil, were administered to the abdominal cavity of nude mice (Shanghai Laboratory Animal Center, Chinese Academy of Sciences, Shanghai, China) of 6 weeks old by intraperitoneal injection for 15 sec. Twenty hours after carbon tetrachloride administration, liver tissues from nude mice were fixed in 4% PFA, embedded in paraffin, and sectioned at 5 μm thickness. The sections were stained with hematoxylin and eosin (H&E), and the degrees of liver necrosis were determined under the macroscope and microscope.

### Transplantation of human SSC line and mouse liver mesenchymal cells under renal capsules of nude mice

Transplantation of human SSC line and mouse liver mesenchymal cells under renal capsule of nude mice was performed pursuant to the method described previously [38]. Briefly, 2 × 10^6^ cells were recombined at a ratio with 85% of human SSC line and 15% of liver mesenchymal cells (hepatic stellate cells, endothelial cells, and Kupffer cells) in 15 ml tube. Human SSC line without liver mesenchymal cells served as a control. The resultant cell recombinants were placed in 200 μl of PBS with 3 mg/ml collagen type I (10 μl with 10^5^ cells per graft), incubated at 37°C for 1 h–1.5 h to allow collagen gelation, and cultured with DMEM/F12 medium. After incubation with DMEM/F12 overnight at 37°C and 5% CO_2_ incubator, the cells in collagen gel were grafted under the renal capsule of 6-week-old nude mice according to the method described previously [39]. Mice with liver injury caused by carbon tetrachloride were anaesthetized by 0.01 ml/g boy weight of Avertin. The skin of the mice was sterilized by 75% of alcohol and 10 mm of inclusions were made in mouse abdomen to expose the kidneys. The capsules of kidneys were open and transplanted with 10 μl graft with 10^5^ of human SSC line and mouse liver mesenchymal cells. The capsules were sutured, and each kidney was transplanted with two cell grafts. Four weeks after implantation, grafts were harvested and analyzed for eGFP expression and hallmarks of hepatocytes by immunohistochemistry as described below. The experiments were performed three times. This study was approved by The Institutional Ethical Review Committee of Ren Ji Hospital, Shanghai Jiao Tong University School of Medicine.

### Histological examination

Various kinds of organs and tissues, including liver, spleen, heart, lung, brain, and kidney, from recipient mice, normal nude mice, and liver injury mice were fixed in 4% PFA, embedded in paraffin, and sectioned at 5 μm thickness. The sections were stained with hematoxylin and eosin, and they were observed under a microscope.

### Immunohistochemistry

To assess whether human SSC line could convert to hepatocytes *in vivo*, immunohistochemistry was conducted in term of the method as described previously [33]. Briefly, grafts and liver tissues form recipient mice were fixed in 4% PFA, dehydrated through graded alcohols, embedded in paraffin, and sectioned at 5 μm thickness. The sections were deparaffinized and rehydrated, and antigen retrieval was performed in 10 mM sodium citrate buffer solution for 20 min at 96°C. Endogenous peroxidase activity was quenched by incubation with 3% hydrogen peroxide. After permeabilization with 0.4% Triton X-100 and blocking with 5% donkey serum (Maibio), the sections were incubated with primary antibodies, including ALB (albumin) (Santa Cruz) and AAT (alpha-1antiproteinase) (Bethyl), in a humidified chamber overnight at 4°C. After washes three times with PBS, the sections were incubated with horse radish peroxidase-conjugated secondary antibody for 1 hour at room temperature and followed by 3, 3-diaminobenzidine (DAB) as a substrate. The dilutions, resources, and companies of primary and secondary antibodies for immunohistochemistry were shown in Table 2. After immunostaining, sections were counterstained with hematoxylin and examined under a light microscope (Nikon Eclipse Ti-S, Nikon Corporation, Tokyo, Japan).

For immunofluorescence, similar procedures were performed except for peroxidase quenching. The primary antibodies were CK18 (Santa Cruz), CYP1A2 (Santa Cruz), human nuclear antigen (HumNuc) (Millipore), GPR125 (Abcam), and VASA (Abcam), and incubated in a humidified chamber overnight at 4°C. The secondary antibodies were rhodamine-conjugated IgG and FITC-conjugated IgG and incubated for 1 hour at room temperature. The dilutions, resources, and companies of primary and secondary antibodies for immunohistochemistry were shown in Table 2. Replacement of primary antibody with PBS was utilized as a negative control. DAPI was used to stain cell nuclei and the sections were observed the epifluorescence using fluorescence microscope (Nikon Eclipse Ti-S, Nikon Corporation, Tokyo, Japan).

### Western blots

The grafts were minced thoroughly and lysed with RIPA buffer (Santa Cruz) on ice. Western blots were performed according to the protocol as described previously [33]. Briefly, the concentration of total proteins was measured first by BCA kit (Dingguo Company, China). Twenty micrograms of cell lysates were resolved by SDS-PAGE (Bio-Rad Laboratories, Richmond, CA). The chosen antibody included CYP1A2 (Santa Cruz), AAT (Bethyl), AFP (alpha-fetoprotein) (Santa Cruz), ALB (Santa Cruz), PCNA (Santa Cruz), GPR125 (Abcam), and ACTB (Proteintech). The dilutions, resources, and companies of primary and secondary antibodies for Western blots were shown in Table 2. After extensive washes in PBS, the blots were detected by chemiluminescence (Chemi-Doc XRS, Bio-Rad, Hercules, CA).

### Statistical analysis

All data were obtained from at least three independent experiments. Statistical analyses were determined using analysis of variance (ANOVA) and a 2-tailed *t*-test, and *P* values of less than 0.05 (*p* < 0.05) were considered statistically significant.
